# Supplementary material for: Absolute and Relative Reliability of Spatiotemporal Gait Characteristics Extracted from an Inertial Measurement Unit among Senior Adults Using a Passive Hip Exoskeleton: A Test–Retest Study
Source: Sensors (Basel). 2024 Aug 12;24(16):5213. doi: 10.3390/s24165213 (PMC11360760; doi:10.3390/s24165213)
Supplement: Supplementary file 1 [file sensors-24-05213-s001.zip › sensors-3046543-supplementary.pdf]

Supplementary S1

**Check list of items - Guidelines for Reporting Reliability and Agreement Studies (GRRAS).**		**Page No**
**TITLE AND ABSTRACT**	1. The abstract identifies that absolute and relative reliability was investigated.	P3, line 13-16.
**INTRODUCTION**	2. The measurement devices and the experimental procedures are mentioned explicitly.	P5, line 39 to P8, Line 124
	3. The subject population is specified.	P5, line 50-53 and P6, line 72-73
	4. Not applicable (information on rater population).	NA
	5. Information about reliability and rationale for the study are provided.	P6, line 61-66
**METHODS**	6. We explain how the sample size was chosen.	P5, line 50-53 and P6, line 72-73
	7. We explain the sampling method.	P7, line 102-108
	8. We describe the measurement process including time interval between repeated measurements and availability of clinical information.	P6, line 80-104 and S2 Appendix
	9. The measurements were made over two experimental sessions.	P6, line 81
	10. The statistical analysis is described.	P9, Line 130-151
**RESULTS**	11. We state the actual number of rater (one) and the included subjects as well as the number of replicate measurements.	NA

	12. We describe the sample characteristics of the subjects.	P6, line 72-73
	13. We report estimates of reliability and agreement including measures of statistical uncertainty.	P10, Line 153 to P11, Line:175
**DISCUSSION**	14. We discuss the practical relevance of results.	P11, Line 178 to P13, Line: 230
**AUXILIARY MATERIAL**	15. We provide supplementary materials	S2: Appendix

Supplementary S2

The Mobilise-D consortium algorithm was adapted to analyze our experimental data set (no gyroscope data) and to extract cadence, step length and gait speed. The following modifications were made to the code:

1. The algorithm requires gyroscope data, requirement bypassed by commenting those specific MATLAB code lines using *%*.
2. The algorithm applied a Hilbert envelope to determine sections of data where walking occurred. We added to this section that the code would only analyze further the walking forward sections and not the walk back sections (see Figure 1) by manually inputting the sequences into MATLAB using the function *input*.
3. The algorithm has several gait trained models from groups with Parkinson's disease, multiple sclerosis, and healthy adults. The model selected was asymptomatic healthy senior adults in our case [1].
4. The position of the IMU is used as a constant for the estimation of step length and was set to the height of the participant multiplied by 0.53 in agreement with ergonomics body chart [2].
5. The algorithm estimated stride length, which was defined as twice the step length, according to the algorithm output description. Therefore, we divided the stride length by two to estimate walking step length.
6. As the algorithm did not estimate walking speed, we added this output as a product between cadence and step length divided by 60 [3].
7. In this study, the outcome measures were the mean and standard deviation of cadence, step length, and walking speed averaged over five trials as well as the number of steps performed during for each walk trial.

Supplementary S2
Code modifications

+

References

[1] M. E. Micó-Amigo, T. Bonci, A. Paraschiv-Ionescu, M. Ullrich, C. Kirk, A. Soltani, A. Küderle, E. Gazit, F. Salis and L. Alcock, "Assessing real-world gait with digital technology? Validation, insights and recommendations from the Mobilise-D consortium," *Journal of NeuroEngineering and Rehabilitation,* vol. 20, *(1),* pp. 1-26, 2023.

[2] S. Del Din, A. Godfrey and L. Rochester, "Validation of an accelerometer to quantify a comprehensive battery of gait characteristics in healthy older adults and Parkinson's disease: toward clinical and at home use," *IEEE Journal of Biomedical and Health Informatics,* vol. 20, *(3),* pp. 838-847, 2015.

[3] R. B. Dale, "Clinical gait assessment," in *Physical Rehabilitation of the Injured Athlete*Anonymous Elsevier, 2012, pp. 464-479.
